# Development of a multiplex loop-mediated isothermal amplification (LAMP) method for differential detection of *Mycobacterium bovis* and *Mycobacterium tuberculosis* by dipstick DNA chromatography

**DOI:** 10.1128/spectrum.02421-24

**Published:** 2025-04-30

**Authors:** Mwangala L. Akapelwa, Thoko F. Kapalamula, Lavel C. Moonga, Precious Bwalya, Eddie S. Solo, Joseph Y. Chizimu, Jeewan Thapa, Kyoko Hayashida, Bernard M. Hang'ombe, Musso Munyeme, Aki Tamaru, Takayuki Wada, Shiomi Yoshida, Takuya Kodera, Mistuo Kawase, Stephen V. Gordon, Keiko Yamada, Chie Nakajima, Yasuhiko Suzuki

**Affiliations:** 1 Division of Bioresources, Hokkaido University International Institute for Zoonosis Control, Sapporo, Japan; 2 Department of Pathobiology, Faculty of Veterinary Medicine, Lilongwe University of Agriculture and Natural Resources 128320, Lilongwe, Central Region, Malawi; 3 School of Veterinary Medicine, University of Zambia 247512, Lusaka, Lusaka Province, Zambia; 4 Department of Pathology and Microbiology, University Teaching Hospital, Lusaka, Zambia; 5 Zambia National Public Health Institute, Ministry of Health 108232 , Lusaka, Lusaka Province, Zambia; 6 Division of collaboration and education, Hokkaido University International Institute for Zoonosis Control, Sapporo, Japan; 7 Africa Center of Excellence for Infectious Diseases of Humans and Animals, University of Zambia 108234, Lusaka, Lusaka Province, Zambia; 8 Hokkaido University, Institute for Vaccine Research and Development 12810, Sapporo, Hokkaido Prefecture, Japan; 9 Department of Microbiology, Osaka Institute of Public Health 91397 , Osaka, Osaka Prefecture, Japan; 10 Graduate School of Human Life and Ecology, Osaka Metropolitan University 12936, Osaka, Osaka Prefecture, Japan; 11 Clinical Research Center, National Hospital Organization Kinki-Chuo Chest Medical Center 73782, Sakai, Osaka Prefecture, Japan; 12 Tohoku Bio-Array Co., LTD, Sendai, Japan; 13 School of Veterinary Medicine and UCD Centre for Experimental Pathogen Host Research, University College Dublin 8797, Dublin, Leinster, Ireland; FIND, Geneva, Switzerland

**Keywords:** *M. bovis*, *M. tuberculosis*, simultaneous detection, multiplex LAMP, dipstick chromatography, biovar differentiation

## Abstract

Although human tuberculosis (TB) caused by *Mycobacterium bovis* is clinically, pathologically, and radiologically indistinguishable from *Mycobacterium tuberculosis*-caused TB, *M. bovis* is innately resistant to pyrazinamide, a key first-line drug effective against *M. tuberculosis*. The rapid differentiation of these two biovars is therefore of high clinical and epidemiologic importance. Most current molecular tools in resource-limited settings identify mycobacteria only to the *M. tuberculosis* species (MTB) level. In this study, we report a multiplex loop-mediated isothermal amplification (LAMP) method coupled with dipstick chromatography for the rapid and easy differential detection of *M. bovis* and *M. tuberculosis*. The assay was optimized and validated using 143 isolates comprising six MTB reference strains, 50 *M*. *bovis* isolates, 58 *M*. *tuberculosis* isolates, 24 non-tuberculous mycobacterial (NTM) strains, and five other respiratory pathogens. The multiplex LAMP correctly detected MTB and distinguished between *M. tuberculosis* and *M. bovis* simultaneously with sensitivities of 500 fg and 1 pg DNA, respectively, within 60 min, and the results were visualized by dipstick chromatography within 10 min. The assay was specific in that no major respiratory pathogens tested, including NTM strains, were positive. The multiplex dipstick LAMP assay is therefore a useful and accurate low-cost method for the differential identification of *M. bovis* and *M. tuberculosis*, especially in endemic areas where bovine and human TB coexist. The distinction between *M. bovis* and *M. tuberculosis* can also aid in monitoring the spread of *M. bovis* to humans and allow for correct treatment, which will ultimately contribute to TB control in both humans and animals.

**IMPORTANCE:**

Human tuberculosis caused by *Mycobacterium tuberculosis* and *Mycobacterium bovis* shows similar clinical symptoms; however, the treatment differs because *M. bovis* is inherently resistant to pyrazinamide, a key first-line drug effective against *M. tuberculosis*. Most available molecular tools cannot distinguish the two biovars. This study addresses this gap by introducing a multiplex loop-mediated isothermal amplification (LAMP) method coupled with dipstick chromatography that can simultaneously and differentially detect *M. bovis* and *M. tuberculosis* within 60 min. The LAMP method does not require sophisticated high-cost equipment and can be easily implemented in resource-limited settings. Our LAMP facilitates rapid and accurate tuberculosis diagnosis, enabling appropriate therapeutic agents to be selected in areas where bovine and human tuberculosis coexist. It can also screen for *M. bovis* infection in humans and livestock, providing prevalence data in areas where such information is lacking.

## INTRODUCTION

*Mycobacterium tuberculosis* var. *bovis* (*M. bovis*) and *Mycobacterium tuberculosis* var. *tuberculosis* (*M. tuberculosis*) are the etiological agents of bovine tuberculosis (bTB) and tuberculosis (TB) in animals and humans, respectively. While *M. tuberculosis* causes human TB, *M. bovis* causes TB in a wide range of animal hosts, including humans. This has made bTB one of the most important zoonotic diseases worldwide of public health concern (1). In 2016, WHO published a rough estimate of the global burden of zoonotic TB, with 147,000 new cases and 12,500 deaths annually (2). Although *M. tuberculosis* is the main cause of human TB, an estimated 2.8% of new human TB cases in African countries are reportedly caused by *M. bovis* (3).

Tuberculosis can generally be treated by the WHO-recommended regimen comprising isoniazid (INH), rifampicin (RIF), ethambutol (EMB), and pyrazinamide (PZA). Pyrazinamide is also a key component of drugs recommended for the management of multidrug-resistant (MDR) TB, owing to its ability to act in the acidic environment in which *M. tuberculosis* persists (4, 5). However, human TB caused by *M. bovis* cannot be treated with PZA because *M. bovis* strains are naturally resistant to PZA (6). PZA is known to be the most hepatotoxic of the first-line TB drugs, and its toxicity can be quite severe (4). Therefore, to avoid treatment failures and unnecessary exposure to the potentially toxic drug, there is a need to accurately distinguish *M. bovis* from *M. tuberculosis*, especially in areas where bovine and human TB are endemic and coexist. Furthermore, the distinction of *M. bovis* from *M. tuberculosis* can assist in monitoring the zoonotic spread of *M. bovis* to humans, which is important in the formulation of effective control measures (7).

Despite the clinical implications of *M. tuberculosis* biovar differentiation, it is not routinely performed, partly due to the close genetic relatedness of *M. bovis* and *M. tuberculosis,* which makes definitive detection to the biovar level both difficult and time-consuming. Conventional culture and biochemical tests have previously been employed in some studies to differentiate *M. bovis* from *M. tuberculosis*. Unfortunately, these methods have the disadvantage of being laborious, slow, and unreliable due to the emergence of strains with variable and intermediate biochemical patterns (7). Nucleic acid amplification tests (NAATs) are a reliable approach for *M. bovis* from *M. tuberculosis* differentiation. However, these methods have certain disadvantages, such as high costs of equipment and maintenance, among others, which makes them unfavorable for routine use in resource-limited settings (8, 9).

On the other hand, loop-mediated isothermal amplification (LAMP) is a widely accepted, rapid, low-cost, isothermal DNA amplification technique that utilizes *Bst* DNA polymerase with strand displacement activity (10). Several highly sensitive LAMP methods have been developed for the detection of *M. tuberculosis* (11–13), including a method we previously developed for *M. bovis* detection (14). However, these LAMP systems are limited to detecting a single target due to the use of turbidity and/or fluorescence, which does not allow for differentiation of multiple amplicons. Nevertheless, multiplex LAMP techniques have been established that achieve the simultaneous detection of multiple DNA targets in a single reaction (15). The resultant multiple LAMP products are distinguished by the incorporation of various visualization methods, ranging from fluorophore quencher-based probes to DNA lateral flow dipstick techniques (16–19). Given that the use of fluorescence-based systems requires sophisticated equipment and technical expertise, DNA lateral flow dipstick techniques are a more cost-effective approach (19–21).

Single-stranded tag hybridization chromatographic printed-array strip (STH C-PAS) is a method developed by Tohoku Bio-Array Company Limited (TBA Co., Ltd., Miyagi, Japan), which allows the discrimination of multiple amplified products simultaneously by the principle of DNA-DNA hybridization and DNA lateral flow dipstick (20, 22). In STH C-PAS, target DNA is amplified with a biotin-labeled primer and a unique single-stranded tagged primer. The resultant biotin-labeled amplicons are mixed with streptavidin-coated blue latex beads that form a hybrid due to the strong biotin-streptavidin affinity. When the tip of the dipstick strip (C-PAS) is inserted into the mixture, the amplicon-blue latex hybrids move up through the strip by capillary action and are captured by hybridization to the tag-complementary sequence printed as test lines on the C-PAS (https://www.t-bioarray.com/en_contents/technology.html). The accumulation of the colored amplicons (amplicon-blue latex hybrids) induces a visible blue test line signaling the presence of the target DNA in the sample (23–25). Multiplex DNA signals in a single tube can be easily differentiated and visualized by the C-PAS method within 10 min. Owing to its simplicity, C-PAS has been used as a visualization technology for differential and simultaneous detection of pathogens (21, 23–28) as well as in the food industry (19, 20).

In this report, we describe a low-cost multiplex LAMP-PAS assay for the easy and rapid simultaneous differential detection of *M. bovis* and *M. tuberculosis* and applicable for the surveillance of tuberculosis in livestock and humans. The differentiation of *M. bovis* and *M. tuberculosis* is essential for the proper management of human TB caused by *M. bovis*, while routine surveillance of human and livestock TB can aid in monitoring the transmission dynamics of *M. bovis* from cattle to humans.

## MATERIALS AND METHODS

### Study samples

A total of 143 mycobacterial and non-mycobacterial strains were used in this study comprising six *M. tuberculosis* species (MTB) reference strains, non-tuberculous mycobacteria (NTM) reference strains (*n* = 24), non-mycobacterial respiratory reference strains (*n* = 5), *M. tuberculosis* clinical isolates (*n* = 58), and *M. bovis* tissue isolates (*n* = 50). *M. bovis* BCG Tokyo 172 was provided by the Japan BCG Laboratory (Tokyo, Japan). *M. orygis* NepR1 and *M. caprae* EPDC01 were isolated from wild and captive animals and characterized previously (29, 30). Other MTB reference strain samples were provided as extracted and purified DNA by Osaka Institute of Public Health (Osaka, Japan). NTM reference strains were obtained from the Japan Anti-Tuberculosis Association (JATA) Research Institute of Tuberculosis (Tokyo, Japan). Five non-mycobacterial strains were purchased from Biological Resource Center, National Institute of Technology and Evaluation (NBRC) (Chiba, Japan).

Thirty *M. bovis* samples were collected from cattle suspected of TB during routine postmortem at Lilongwe cold storage abattoir in Lilongwe Malawi in 2019, and the isolates grown in Mycobacterium Growth Indicator Tubes (MGIT) (Becton, Dickinson and Company, NJ, USA) were obtained at National Tuberculosis Reference Laboratory, Lilongwe, Malawi (31). Twenty *M. bovis* isolates grown on Ogawa medium (Kyokuto Pharmaceutical Industrial Co., Ltd., Tokyo, Japan) were collected from cattle and wild lechwe antelope from 2004 to 2008 in Zambia (32). Twenty-three *M. tuberculosis* clinical samples grown in MGIT were obtained at the University Teaching Hospital in Lusaka, Zambia during 2014 to 2016 (33). The other 35 *M*. *tuberculosis* isolates were collected in Osaka, Japan, during 2000 to 2009 and grown on Ogawa medium at Osaka Institute of Public Health (34). All the used clinical or tissue isolates were confirmed as *M. tuberculosis* or *M. bovis* by spoligotyping and multiplex PCR reported by Bakshi et al. (9). Details of the isolates used in this study are outlined in Table 1 and Table S1.

**TABLE 1:** Bacteria used in this study to determine specificity of the LAMP-PAS^*d*^

Category	Bacterial species/biovar	Sample ID
MTB reference strains	*Mycobacterium bovis* BCG	Tokyo 172
	*M. tuberculosis*	H37Rv
	*M. africanum*	KK 13-02
	*M. microti*	ATCC 19422
	*M. orygis*	NepR1^*a*^
	*M. caprae*	EPDC01^*b*^
MTB clinical isolates	*M. bovis*	50 isolates^*c*^
	*M. tuberculosis*	58 isolates^*c*^
NTM reference strains	*M. avium*	JATA 51-01
	*M. gastri*	KK 44-01
	*M. gordonae*	JATA 33-01
	*M. intermedium*	JATA 9H-01
	*M. intracellulare*	JATA 52-01
	*M. kansasii*	KK 21-01
	*M. lentiflavum*	JATA 9N-01
	*M. asiaticum*	KK 24-01
	*M. malmoense*	JATA 47-01
	*M. shimodei*	JATA 54-01
	*M. ulcerans*	JATA 43-02
	*M. marinum*	JATA 22-01
	*M. celatum*	JATA 9L-01
	*M. xenopi*	JATA 42-01
	*M. simiae*	JATA 23-01
	*M. scrofulaceum*	JATA 31-01
	*Mycolicibacter nonchromogenicus*	JATA 45-01
	*Mycolicibacillus trivialis*	JATA 50-01
	*Mycobacteroides chelonae*	JATA 62-01
	*Mycobacteroides abscessus*	JATA 63-01
	*Mycolicibacterium fortuitum*	JATA 61-01
	*Mycolicibacterium mucogenicum*	JATA 9P-01
	*Mycolicibacterium peregrinum*	JATA 61-02
	*Mycolicibacterium smegmatis*	JATA 64-01
Non-mycobacteria	*Pseudomonas aeruginosa*	NBRC 12689
	*Staphylococcus aureus*	NBRC 100910
	*Mycoplasma pneumoniae*	NBRC 14401
	*Streptococcus pneumoniae*	NBRC 102642
	*Klebsiella pneumoniae*	NBRC 3318

^*a*^ Reference 29

^*b*^ Reference 30

^*c*^ More information in Table S1.

^*d*^ JATA: JATA: Japan Anti-Tuberculosis Association, KK: Kekkaku Kenkyusho (= JATA).

### Sample storage and DNA extraction

Reference strains were suspended in 20% glycerol and stored at −80°C at Hokkaido University, Japan. Clinical and tissue isolates were stored in the collaborator’s laboratories, and only extracted DNA samples were transferred to Japan. Genome DNA from the reference strains and Osaka isolates was extracted and purified by the bead-beating method, followed by chloroform purification and ethanol precipitation as previously described (35). For clinical and tissue isolates cultured in liquid MGIT medium, 500 µL of the medium was taken in a cryotube, and crude DNA was extracted by repeated boiling at 95°C for 15 min and freezing at −30°C. For isolates on solid medium, a spoonful colony was suspended in 500 µL of TE buffer in a cryotube, and crude DNA was extracted by repeated boiling and freezing as described above. The final bacterial suspension was centrifuged, and the supernatant was used as the sample. All the extracted DNA samples were kept at −30°C until use.

### Multiplex LAMP primers

Two sets of specific primers targeting *M. bovis* RD4 (14) and the 16S rRNA gene of MTB (12) were synthesized based on our previously reported LAMP protocols. The *M. bovis* RD4 primers were specific for the RD4 deletion of *M. bovis,* while 16S rRNA primers targeted all MTB biovars, which include *M. bovis*. Hence, *M. bovis* could be positively identified with both primer sets, while other MTB biovars including *M. tuberculosis* could be identified only by the second primer set. The multiplex LAMP reaction was comprised of two sets of six LAMP primers per target (a total of 12 primers). Two primers from each set underwent some modifications at the 5′ terminal ends. Specifically, the backward loop primer (LB) of the *M. bovis* RD4 LAMP primer set and the forward loop primer (LF) of the MTB 16S rRNA primer set were tagged with a carbon spacer and a unique tag sequence complementary to the specific oligos printed on the C-PAS (TBA Co., Ltd.) (22). Furthermore, the forward loop primer (LF) and backward inner primer (BIP) targeting *M. bovis* RD4 and MTB 16S rRNA, respectively, were labeled with biotin at the 5′-terminal (TBA Co., Ltd.). No modifications were made to any of the other primers as outlined in Table 2. All primers without modification were synthesized by Life Technologies Japan Ltd. (Tokyo, Japan).

**TABLE 2:** Primers used in this study

Target pathogen	Target gene name	Primer name [Ref]	Primer sequence name	Primer sequence
*M. bovis*	RD4	*M. bovis* RD4 (14)	RD4-F3	5′′-GCCGCTCCCAAAAATTACCA-3′
			RD4-B3	5′-GACGCTACTACGGCACGG-3′
			RD4-FIP	5′-AGGCCACTCCAAGAGTGTTGCGTGACGCCTTCCTAACCAGA-3′
			RD4-BIP	5′-GCGCGGGCGTACCGGATATGCGCCCCGTAGCGTTA-3′
			RD4-LF-Bio	5′-Bio-CTTCTGCACGACTACGGCT-3′
			RD4-LB-TagF1	5′-TagF1-AGCCATTTTTCAGCAATTTCTCAG-3′
MTB	16S rRNA	MTBC 16S rRNA (12)	16 S-F3	5′-CTGGCTCAGGACGAACG-3′
			16S-B3	5′-GCTCATCCCACACCGC-3′
			16S-FIP	5′-CACCCACGTGTTACTCATGCAAGTCGAACGGAAAGGTCT-3′
			16S-BIP-Bio	5′-Bio-TCGGGATAAGCCTGGACCACAAGACATGCATCCCGT-3′
			16S-LF-TagF2	5′-TagF2-GTTCGCCACTCGAGTATCTCCG-3′
			16S-LB	5′-GAAACTGGGTCTAATACCGG-3′

^*a*^ Tagged and biotin-labeled primers are indicated by TagF1-, TagF2- and Bio-, respectively. The nucleotide sequence of TagF1 and TagF2 is unique (TBA Co., Ltd.).

### Multiplex LAMP amplification

The LAMP mixture was the same as previously reported (12, 14)[ however, the LAMP primers were diluted in varying proportions to achieve maximum sensitivity and specificity. The multiplex LAMP reaction was performed in 25 µL reaction volumes as previously described (12). Each reaction contained 2 µL of the DNA template, specific concentrations of primers, 0.8 M betaine (Sigma-Aldrich, St Louis, MO, USA), 20 mM Tris-HCl (pH 8.8) (Wako Pure Chemical Industries, Osaka, Japan), 1.25 mM deoxynucleoside triphosphate mix, 10 mM KCl, 10 mM (NH4)_2_SO_4_, 0.1% Tween 20 (Sigma-Aldrich), 6 mM MgSO_4_, and 8 U of *Bst* DNA polymerase (Nippon Gene Co, Ltd, Tokyo, Japan). These reagents were mixed in one tube, and double distilled water (DDW) was added up to a final volume of 25 µL. Thereafter, the mixture was incubated at 66°C in a Loopamp real-time turbidimeter (LA-200; Teramecs Co, Kyoto, Japan) for up to 60 min. The results were considered positive based on the increase in turbidity curves to greater than a threshold of 0.1 according to the manufacturer’s instructions. Extracted DNA from *M. bovis* BCG Tokyo 172 and *M. tuberculosis* H37Rv was used as positive control and DDW as a negative control. The reaction mixture was prepared in a clean room before being transferred to the amplification room, where the DNA templates were added.

### Visualization of multiplex amplicons by dipstick DNA chromatography (C-PAS)

After the multiplex LAMP reaction, the C-PAS F4 membrane strip (TBA Co., Ltd.) was inserted into a 33 µL reaction mix containing 30 µL of developing solution (300 mM NaCl) (TBA Co., Ltd.), 2 µL of streptavidin-coated blue latex suspension (TBA Co., Ltd.), and 1 µL of LAMP product. The strip was incubated for 10 min at room temperature, after which the results were interpreted. A positive test was indicated by the appearance of a blue line on the C-PAS strip test position signaling the presence of the amplified target DNA sequence tagged with the complementary oligonucleotide and biotin. The best combination of modified primers and the ratio of each primer were determined from the coloration of the bands of interest. The LAMP amplification and the visualization by dipstick chromatography were performed in the post-amplification room, which is separate from the pre-amplification clean room.

### Specificity analysis

The multiplex LAMP specificity was evaluated against MTB reference strains, NTM strains, non-mycobacterial respiratory strains, *M. tuberculosis* clinical isolates, and *M. bovis* tissue isolates (Table 1; Table S1) in comparison with the multiplex PCR assay targeting RD4 described by Bakshi et al. (9). The multiplex PCR procedure was slightly modified. Briefly, 4 µL 5× Go Taq buffer (Promega Co., WI, USA); 0.8 µL of 25 mM MgCl_2_; 0.2 µL of 25 mM each dNTP mix; 2 µL of 5 M betaine, 0.5 µL of 10 µM each primer; common forward primer - CBS1 (5′-TTCCGAATCCCTTGTGA-3′); *M. bovis-*specific reverse primer—CBS2 (5′-GGAGAGCGCCGTTGTA-3′), and *M. tuberculosis-*specific reverse primer—CBS3 (5′-AGTCGCCGTGGCTTCTCTTTTA-3′); 0.1 µL of 5 U/µL GoTaq DNA polymerase (Promega Co.); 1 µL of template DNA; and finally DDW to make up to a final volume of 20 µL. The cycling parameters consisted of an initial denaturation at 94°C for 5 min, followed by 30 cycles of denaturation at 94°C (1 min), annealing at 52°C (1.5 min), and extension at 72°C (1 min), with a final elongation step at 72°C for 5 min. The amplification products were analyzed by gel electrophoresis at 100 V for 20 min. The predicted PCR products were 168 bp (*M. bovis*) and 268 bp (MTB other than *M. bovis*).

### Evaluation of the limit of detection (LoD)

The LoD of our multiplex LAMP system was evaluated using serially diluted *M. bovis* BCG Tokyo 172 and *M. tuberculosis* H37Rv genomic DNA, ranging from 250 pg/µL to 25 fg/μL (500 pg, 50 pg, 5 pg, 2 pg, 1 pg, 500 fg, 200 fg, 100 fg, and 50 fg/reaction). The DNA concentration was measured using Qubit 3 Fluorometer (Thermo Fisher Scientific, MA, USA) according to the manufacturer’s instructions. Reactions were performed multiple times as shown in Table S2.

## RESULTS

### Rapid differential detection of *M. bovis* and *M. tuberculosis* by multiplex LAMP

Multiplex LAMP reaction was optimized for *M. bovis* and *M. tuberculosis* detection by utilizing a Loopamp real-time turbidimeter coupled with C-PAS. The following concentrations were selected for each primer after examining various concentrations: 1.6 µM FIP/BIP, 0.8 µM LF/LB, 0.2 µM F3/B3 of RD4 LAMP primer set, and for MTB LAMP primer set, 0.8 µM FIP/BIP, 0.4 µM LF/LB, and 0.1 µM F3/B3. The optimal incubation temperature and time were 66°C and 60 min, respectively. The multiplex LAMP reaction results were ready within 60 min, while visualization of results by dipstick chromatography took 10 min.

### Specificity of the multiplex LAMP

**Fig 1:**
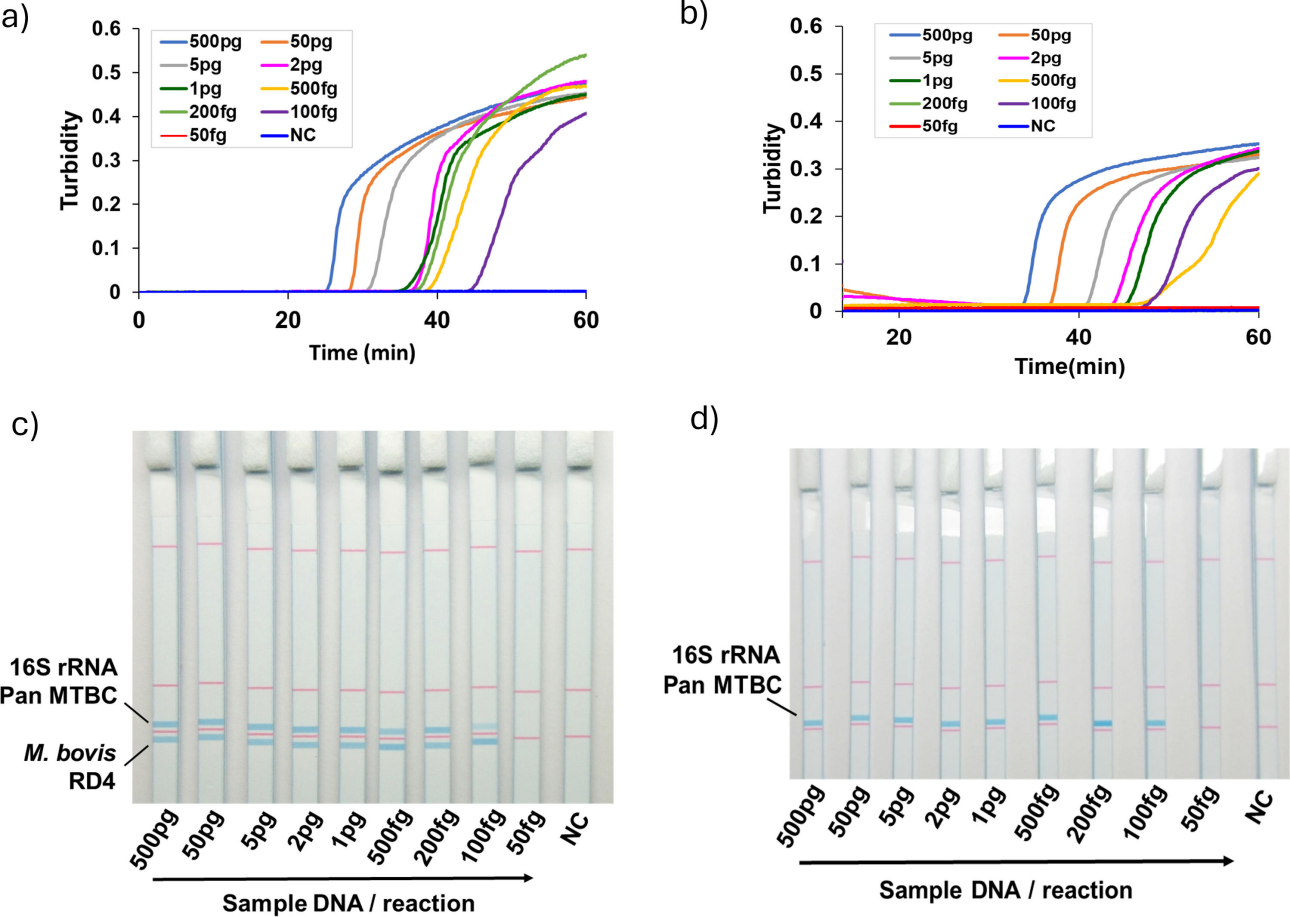
Multiplex LAMP amplification and discrimination by dipstick DNA chromatography (C-PAS). (**a**,** b**) Multiplex LAMP results for *M. bovis* BCG (**a**) and *M. tuberculosis* H37Rv (**b**) detection by rising curves from the turbidimeter. (**c**,** d**) Results of *M. bovis* BCG (**c**) and *M. tuberculosis* H37Rv (**d**) detection by multiplex LAMP coupled with C-PAS. Upper band is for the detection of MTB 16S rRNA, and the lower band is for the detection of *M. bovis* specific RD4. *M. bovis* shows two bands, while other MTB shows only one upper band. NC: negative control

The specificity of the multiplex LAMP assay was evaluated with strains shown in Table 1 and Table S1. In all cases, reaction mixtures with the template DNAs from *M. bovis* isolates showed two positive bands on the C-PAS indicating the presence of both targets (*M. bovis-*specific RD4 and MTB 16S rRNA) in the tested samples. On the other hand, all reactions with the DNAs from *M. tuberculosis* isolates and MTB strains other than *M. bovis* consistently showed only one positive band on the C-PAS, which signaled the detection of the target MTB 16S rRNA in the test samples (Fig. 1 and Table 3; Table S1). All the results were concordant with those of the multiplex PCR (9), namely, *M. bovis* showed a band of 168 bp, and MTB other than *M. bovis* was 268 bp. While the assay successfully detected all *M. bovis*, *M. tuberculosis,* and MTB strains, no positive test bands, i.e., no amplification occurred, were observed with any of the non-mycobacterial strains tested. Of the 24 NTM tested, *M. marinum, M. ulcerans,* and *M. asiaticum* showed a positive band for the 16S rRNA gene (Table 3). A multiple alignment comparison of the target region of the 16S rRNA gene of these three species indicated high sequence identity to that of MTB (Supplementary Figure). These findings indicate that the two sets of primers utilized in this study are highly specific for the detection of *M. bovis* and MTB, though there were a few exceptions.

**TABLE 3:** Summary of multiplex LAMP-PAS results across all bacteria tested in comparison to established MTB-discrimination multiplex PCR

	Multiplex LAMP-PAS			Multiplex PCR (9)		
	Positive			Positive		
	MTB (16S)	*M. bovis* (RD4)	Negative	MTB^*a*^ (268 bp)	*M. bovis* (168 bp)	Negative
*M. bovis* ^*b*^	51	51	0	0	51	0
*M. tuberculosis* ^*b*^ *M. africanum* *M. microti* *M. orygis* *M. caprae*	59 1 1 1 1	0 0 0 0 0	0 0 0 0 0	59 1 1 1 1	0 0 0 0 0	0 0 0 0 0
NTM^*c*^	0	0	21	na^*d*^	na	na
*M. ulcerans*	1	0	0			
*M. marinum*	1	0	0			
*M. asiaticum*	1	0	0			
Non-mycobacteria	0	0	5	na	na	na

^*a*^ MTB other than *M. bovis.*

^*b*^ Fifty field isolates (*M. bovis*)/58 clinical isolates (*M. tuberculosis*) and one reference strain.

^*c*^ Nontuberculous mycobacteria (NTM) other than *M. ulcerans*, *M. marinum*, and *M. asiaticum.*

^*d*^ na: not applicable.

### LoD of the multiplex LAMP

To estimate the LoD of the multiplex LAMP assay, 10-fold serial dilutions and additional concentrations of each of M. *bovis* BCG Tokyo 172 and *M. tuberculosis* H37Rv genomic DNA were used. The multiplex LAMP was able to detect as low as 500 fg of both *M. bovis* and *M. tuberculosis* genomic DNA per reaction within 60 min and correctly differentiated them when the concentration of DNA was 1 pg/reaction or higher (Fig. 1; Table S2). The amplicons could be easily differentiated by C-PAS in 10 min (Fig. 1C and D). Since concentrations lower than 500 fg/reaction were sometimes not detected as positive and one of the two bands missed in BCG samples, we decided to stop the incubation at 60 min and 1 pg/reaction was set as the differential detection limit.

## DISCUSSION

*Mycobacterium tuberculosis* is known to be the main cause of human tuberculosis; however, 10% to 30% of human TB cases in some African countries are reportedly caused by *M. bovis* (3). Despite this, *M. bovis* differentiation is still not routinely performed due to diagnostic limitations, risking inappropriate case management and poor treatment outcomes. Therefore, assays that can accurately distinguish between these mycobacteria will improve treatment regimens and the establishment of adequate public health control measures. Here, we report a simple multiplex LAMP coupled with dipstick chromatography that allows the rapid identification of *M. bovis* and other MTB. This multiplex LAMP method is advantageous as it can easily achieve simultaneous amplification of multiple target sequences in a single reaction, which saves time and effort.

While many LAMP methods have been reported to specifically detect *M. tuberculosis* or MTB (11, 12, 36), there are few methods to specifically detect *M. bovis*, including PCR methods (9, 14). This is because there are virtually no major variations, such as genes or genomic regions unique to *M. bovis*, that would allow for differentiation from other MTB strains (37). MTB strains do not undergo horizontal gene transfer, with the phylogeny marked by genomic deletions, making it difficult to find genomic regions that are retained only by a particular biovar. Several biovar-specific point mutations have been reported across the MTB; however, aside from PCR methods, current technology has not been able to reproducibly identify these point mutations using isothermal amplification methods (25).

Based on the above, presuming the use of field-available isothermal amplification methods, the most reliable *M. bovis* identification region to date is RD4, which was reported by Gordon et al. in 1999 (37, 38). This region is absent in all *M. bovis* strains known to date, and its length of 12.7 kbp is sufficient to prevent nonspecific amplification in LAMP methods with primer sets designed across the deletion site (14, 37). On the other hand, the 16S ribosomal RNA gene is the reference gene for bacterial species identification, and the variable region we have employed is an ideal target for MTB identification as it is distinguishable from other bacterial species, yet perfectly matched within MTB strains (12). In the present study, three NTM species, *M. marinum, M. ulcerans,* and *M. asiaticum*, showed positive results in the detection of MTB 16S rRNA gene due to the high similarity of the sequence to that of MTB in the region (Fig. S1). However, since the pathogenesis and epidemiology of *M. marinum* and *M. ulcerans* are different from those of MTB (39, 40) and *M. asiaticum* is rarely isolated from clinical specimens (41), we do not believe this will be a cause for confusion in the species determination of MTB. Although the LoD of the multiplex LAMP method was slightly higher than that of each LAMP method alone (12, 14), 1 pg of DNA is equivalent to 200 MTB bacilli, which is sensitive enough for detection while discriminating between the biovars.

The LAMP is an excellent isothermal amplification method that can be achieved with a single enzyme, *Bst* polymerase (10). However, using amplification alone to call positive or negative results has the drawback that nonspecific amplification cannot be distinguished. Although there have been several ways to identify two or more amplifications occurring simultaneously, such as using a fluorescence-quenching system (16, 18), the simplest and most practical method would be to use a lateral flow strip and capture the amplifications in separate bands. In this method, only amplified products sandwiched between two specific sequences are detected, thus avoiding detection of nonspecific amplification. Several researchers have published methods using immunochromatography; however, their stability and inexpensive mass production remain questionable due to the use of antibodies as capturing agents (42, 43). Since STH C-PAS uses single-stranded DNA as the capture, the target can be captured by simply adding a complementary sequenced DNA tag to it. Like DNA arrays, multiple captures can be placed on the same strip, allowing for future applications such as additional targets (19–21, 23–28).

A drawback of the current study is that it did not use specimens collected directly from patients’ sputum or bovine TB lesions prior to culture. Since such specimens are not available in bovine TB-free countries like Japan, we plan to bring this system to endemic countries and conduct additional testing using specimens obtained from hospitals and slaughterhouses. In addition, the cold chain problem needs to be solved in order to implement the project in rural areas where infrastructure development has not caught up and cooling systems are not available. Since both LAMP systems used in this study have been successfully made into dried LAMP systems individually, we plan to dry both systems in a mixed state to confirm their stability and reproducibility (44–46). Once this multiplex-LAMP system can be successfully dried, it can be implemented using only a water bath.

Presently, the true burden of TB due to *M. bovis* remains largely unknown. This multiplex LAMP-PAS can be applied as an epidemiological tool to investigate the prevalence of TB caused by *M. bovis*. This assay can also be used for the general detection of MTB members, including *M. tuberculosis,* which are susceptible to PZA. Most importantly, the rapid differentiation of *M. bovis* and *M. tuberculosis* will allow the administration of suitable treatment regimens and the formulation of appropriate public health control measures.

In conclusion, we have developed a simple and rapid multiplex LAMP-PAS system for the simultaneous and differential detection of *M. bovis* and *M. tuberculosis*. The accuracy, ease, and low-cost qualities of this assay make it suitable for use at point-of-care settings, especially in resource-poor countries, which have high tuberculosis burdens in both humans and animals. The rapid and accurate differential diagnosis of *M. bovis* and *M. tuberculosis* by this multiplex LAMP-PAS technique will facilitate early diagnosis, which is critical for the management and control of tuberculosis in both humans and animals.

## SUPPLEMENTAL MATERIAL

Supplemental material spectrum.02421-24-s0001.pdf Sensitivity results and sequence alignment.

Table S1 spectrum.02421-24-s0002.xlsx Used samples.

ASM does not own the copyrights to Supplemental Material that may be linked to, or accessed through, an article. The authors have granted ASM a non-exclusive, world-wide license to publish the Supplemental Material files. Please contact the corresponding author directly for reuse.

## References

[1] Taye H, Alemu K, Mihret A, Wood JLN, Shkedy Z, Berg S, Aseffa A. 2021. Global prevalence of Mycobacterium bovis infections among human tuberculosis cases: systematic review and meta-analysis. Zoonoses Public Health 68:704–718. doi:10.1111/zph.12868 34169644 PMC8487997

[2] World Health Organization. 2017. Roadmap for zoonotic tuberculosis. Available from: https://iris.who.int/bitstream/handle/10665/259229/9789241513043-eng.pdf?sequence=1

[3] Müller B, Dürr S, Alonso S, Hattendorf J, Laisse CJM, Parsons SDC, van Helden PD, Zinsstag J. 2013. Zoonotic Mycobacterium bovis-induced tuberculosis in humans. Emerg Infect Dis 19:899–908. doi:10.3201/eid1906.120543 23735540 PMC4816377

[4] World Health Organization. 2014. Companion handbook to the WHO guidelines for the programmatic management of drug-resistant tuberculosis. Available from: https://apps.who.int/iris/bitstream/handle/10665/130918/?sequence=1 25320836

[5] Shi W, Zhang X, Jiang X, Yuan H, Lee JS, Barry CE 3rd, Wang H, Zhang W, Zhang Y. 2011. Pyrazinamide inhibits trans-translation in Mycobacterium tuberculosis. Science 333:1630–1632. doi:10.1126/science.1208813 21835980 PMC3502614

[6] Konno K, Feldmann FM, McDermott W. 1967. Pyrazinamide susceptibility and amidase activity of tubercle bacilli. Am Rev Respir Dis 95:461–469. doi:10.1164/arrd.1967.95.3.461 4225184

[7] Allix-Béguec C, Fauville-Dufaux M, Stoffels K, Ommeslag D, Walravens K, Saegerman C, Supply P. 2010. Importance of identifying Mycobacterium bovis as a causative agent of human tuberculosis. Eur Respir J 35:692–694. doi:10.1183/09031936.00137309 20190335

[8] Case RJ, Boucher Y, Dahllöf I, Holmström C, Doolittle WF, Kjelleberg S. 2007. Use of 16S rRNA and rpoB genes as molecular markers for microbial ecology studies. Appl Environ Microbiol 73:278–288. doi:10.1128/AEM.01177-06 17071787 PMC1797146

[9] Bakshi CS, Shah DH, Verma R, Singh RK, Malik M. 2005. Rapid differentiation of Mycobacterium bovis and Mycobacterium tuberculosis based on a 12.7-kb fragment by a single tube multiplex-PCR. Vet Microbiol 109:211–216. doi:10.1016/j.vetmic.2005.05.015 16005166

[10] Notomi T, Okayama H, Masubuchi H, Yonekawa T, Watanabe K, Amino N, Hase T. 2000. Loop-mediated isothermal amplification of DNA. Nucleic Acids Res 28:E63. doi:10.1093/nar/28.12.e63 10871386 PMC102748

[11] Iwamoto T, Sonobe T, Hayashi K. 2003. Loop-mediated isothermal amplification for direct detection of Mycobacterium tuberculosis complex, M. avium, and M. intracellulare in sputum samples. J Clin Microbiol 41:2616–2622. doi:10.1128/JCM.41.6.2616-2622.2003 12791888 PMC156570

[12] Pandey BD, Poudel A, Yoda T, Tamaru A, Oda N, Fukushima Y, Lekhak B, Risal B, Acharya B, Sapkota B, Nakajima C, Taniguchi T, Phetsuksiri B, Suzuki Y. 2008. Development of an in-house loop-mediated isothermal amplification (LAMP) assay for detection of Mycobacterium tuberculosis and evaluation in sputum samples of Nepalese patients. J Med Microbiol 57:439–443. doi:10.1099/jmm.0.47499-0 18349362

[13] Kaewphinit T, Ckumdee J, Chansiri K, Santiwatanakul S. 2017. Development and evaluation of a loop-mediated isothermal amplification combined with au-nanoprobe assay for rapid detection of Mycobacterium tuberculosis. Indian J Med Microbiol 35:302–304. doi:10.4103/ijmm.IJMM_15_333 28681828

[14] Kapalamula TF, Thapa J, Akapelwa ML, Hayashida K, Gordon SV, Hang’ Ombe BM, Munyeme M, Solo ES, Bwalya P, Nyenje ME, Tamaru A, Suzuki Y, Nakajima C. 2021. Development of a loop-mediated isothermal amplification (LAMP) method for specific detection of Mycobacterium bovis. PLoS Negl Trop Dis 15:e0008996. doi:10.1371/journal.pntd.0008996 33493196 PMC7833227

[15] Iseki H, Alhassan A, Ohta N, Thekisoe OMM, Yokoyama N, Inoue N, Nambota A, Yasuda J, Igarashi I. 2007. Development of a multiplex loop-mediated isothermal amplification (mLAMP) method for the simultaneous detection of bovine Babesia parasites. J Microbiol Methods 71:281–287. doi:10.1016/j.mimet.2007.09.019 18029039

[16] Tanner NA, Zhang Y, Evans TC. 2012. Simultaneous multiple target detection in real-time loop-mediated isothermal amplification. Biotechniques 53:81–89. doi:10.2144/0000113902 23030060

[17] Wang Y, Wang Y, Lan R, Xu H, Ma A, Li D, Dai H, Yuan X, Xu J, Ye C. 2015. Multiple endonuclease restriction real-time loop-mediated isothermal amplification: a novel analytically rapid, sensitive, multiplex loop-mediated isothermal amplification detection technique. J Mol Diagn 17:392–401. doi:10.1016/j.jmoldx.2015.03.002 26094089

[18] Ball CS, Light YK, Koh CY, Wheeler SS, Coffey LL, Meagher RJ. 2016. Quenching of unincorporated amplification signal reporters in reverse-transcription loop-mediated isothermal amplification enabling bright, single-step, closed-tube, and multiplexed detection of RNA viruses. Anal Chem 88:3562–3568. doi:10.1021/acs.analchem.5b04054 26980448

[19] Takabatake R, Kagiya Y, Minegishi Y, Futo S, Soga K, Nakamura K, Kondo K, Mano J, Kitta K. 2018. Rapid screening detection of genetically modified crops by loop-mediated isothermal amplification with a lateral flow dipstick. J Agric Food Chem 66:7839–7845. doi:10.1021/acs.jafc.8b01765 29949351

[20] Monden Y, Takasaki K, Futo S, Niwa K, Kawase M, Akitake H, Tahara M. 2014. A rapid and enhanced DNA detection method for crop cultivar discrimination. J Biotechnol 185:57–62. doi:10.1016/j.jbiotec.2014.06.013 24954682

[21] Shanmugakani RK, Akeda Y, Yamamoto N, Sakamoto N, Hagiya H, Yoshida H, Takeuchi D, Sugawara Y, Kodera T, Kawase M, Laolerd W, Chaihongsa N, Santanirand P, Ishii Y, Hamada S, Tomono K. 2017. PCR-dipstick chromatography for differential detection of carbapenemase genes directly in stool specimens. Antimicrob Agents Chemother 61:e00067-17. doi:10.1128/AAC.00067-17 28373197 PMC5444138

[22] Niwa K, Oribe A, Okumura H, Shimono M, Nagai K, Hirota T, Yasue H, Kawase M. 2014. Tag/hybridization-based sensitive detection of polymerase chain reaction products. Anal Biochem 464:12–16. doi:10.1016/j.ab.2014.07.010 25051253

[23] Tian L, Sato T, Niwa K, Kawase M, Mayanagi G, Washio J, Takahashi N. 2016. PCR-dipstick DNA chromatography for profiling of a subgroup of caries-associated bacterial species in plaque from healthy coronal surfaces and periodontal pockets. Biomed Res 37:29–36. doi:10.2220/biomedres.37.29 26912138

[24] Kodera T, Yamaguchi T, Fukushima Y, Kobayashi K, Takarada Y, Chizimu JY, Nakajima C, Solo ES, Lungu PS, Kawase M, Suzuki Y. 2021. Rapid and simple detection of isoniazid resistant Mycobacterium tuberculosis utilizing DNA chromatography based technique. Jpn J Infect Dis 74:214–219. doi:10.7883/yoken.JJID.2020.754 33132303

[25] Takarada Y, Kodera T, Kobayashi K, Nakajima C, Kawase M, Suzuki Y. 2020. Rapid detection of rifampicin-resistant Mycobacterium tuberculosis, based on isothermal DNA amplification and DNA chromatography. J Microbiol Methods 177:106062. doi:10.1016/j.mimet.2020.106062 32950563

[26] Sugita-Konishi Y, Kobayashi N, Takasaki K, Kanno T, Itoh M Riztyan Futo S, Asakura H, Taira K, Kawakami Y. 2019. Detection of Sarcocystis spp. and Shiga toxin-producing Escherichia coli in Japanese sika deer meat using a loop-mediatedisothermal amplification-lateral flow strip. J Vet Med Sci 81:586–592. doi:10.1292/jvms.18-0372 30814421 PMC6483920

[27] Moonga LC, Hayashida K, Kawai N, Nakao R, Sugimoto C, Namangala B, Yamagishi J. 2020. Development of a multiplex loop-mediated isothermal amplification (LAMP) method for simultaneous detection of spotted fever group rickettsiae and malaria parasites by dipstick DNA chromatography. Diagnostics (Basel) 10:897. doi:10.3390/diagnostics10110897 33147773 PMC7694008

[28] Hayashida K, Garcia A, Moonga LC, Sugi T, Takuya K, Kawase M, Kodama F, Nagasaka A, Ishiguro N, Takada A, Kajihara M, Nao N, Shingai M, Kida H, Suzuki Y, Hall WW, Sawa H, Yamagishi J. 2023. Field-deployable multiplex detection method of SARS-CoV-2 and influenza virus using loop-mediated isothermal amplification and DNA chromatography. PLoS One 18:e0285861. doi:10.1371/journal.pone.0285861 37192155 PMC10187927

[29] Thapa J, Paudel S, Sadaula A, Shah Y, Maharjan B, Kaufman GE, McCauley D, Gairhe KP, Tsubota T, Suzuki Y, Nakajima C. 2016. Mycobacterium orygis-associated tuberculosis in free-ranging rhinoceros, Nepal, 2015. Emerg Infect Dis 22:570–572. doi:10.3201/eid2203.151929 26890310 PMC4766909

[30] Yoshida S, Suga S, Ishikawa S, Mukai Y, Tsuyuguchi K, Inoue Y, Yamamoto T, Wada T. 2018. Mycobacterium caprae Infection in captive borneo elephant, Japan. Emerg Infect Dis 24:1937–1940. doi:10.3201/eid2410.180018 30226170 PMC6154153

[31] Kapalamula TF, Chizimu J, Belotindos L, Akapelwa M, Shrestha D, Nyenje ME, Munyeme M, Hang’ombe BM, Mkakosya RS, Gordon SV, Nakajima C, Suzuki Y. 2022. Molecular epidemiology of Mycobacterium bovis in central parts of Malawi. Transbound Emerg Dis 69:1577–1588. doi:10.1111/tbed.14127 33900039

[32] Hang’ombe MB, Munyeme M, Nakajima C, Fukushima Y, Suzuki H, Matandiko W, Ishii A, Mweene AS, Suzuki Y. 2012. Mycobacterium bovis infection at the interface between domestic and wild animals in Zambia. BMC Vet Res 8:221. doi:10.1186/1746-6148-8-221 23151267 PMC3514303

[33] Solo ES, Nakajima C, Kaile T, Bwalya P, Mbulo G, Fukushima Y, Chila S, Kapata N, Shah Y, Suzuki Y. 2020. Mutations of rpoB, katG and inhA genes in multidrug-resistant Mycobacterium tuberculosis isolates from Zambia. J Glob Antimicrob Resist 22:302–307. doi:10.1016/j.jgar.2020.02.026 32169686

[34] Tamaru A, Nakajima C, Wada T, Wang Y, Inoue M, Kawahara R, Maekura R, Ozeki Y, Ogura H, Kobayashi K, Suzuki Y, Matsumoto S. 2012. Dominant incidence of multidrug and extensively drug-resistant specific Mycobacterium tuberculosis clones in Osaka Prefecture, Japan. PLoS ONE 7:e42505. doi:10.1371/journal.pone.0042505 22952596 PMC3432034

[35] Suzuki Y, Katsukawa C, Inoue K, Yin Y, Tasaka H, Ueba N, Makino M. 1995. Mutations in rpoB gene of rifampicin resistant clinical isolates of Mycobacterium tuberculosis in Japan. Kansenshogaku Zasshi 69:413–419. doi:10.11150/kansenshogakuzasshi1970.69.413 7751750

[36] Bi A, Nakajima C, Fukushima Y, Tamaru A, Sugawara I, Kimura A, Kawahara R, Hu Z, Suzuki Y. 2012. A rapid loop-mediated isothermal amplification assay targeting hspX for the detection of Mycobacterium tuberculosis complex. Jpn J Infect Dis 65:247–251. doi:10.7883/yoken.65.247 22627308

[37] Mabe L, Muthevhuli M, Thekisoe O, Suleman E. 2024. Accuracy of molecular diagnostic assays for detection of Mycobacterium bovis: a systematic review and meta-analysis. Prev Vet Med 226:106190. doi:10.1016/j.prevetmed.2024.106190 38574490

[38] Gordon SV, Brosch R, Billault A, Garnier T, Eiglmeier K, Cole ST. 1999. Identification of variable regions in the genomes of tubercle bacilli using bacterial artificial chromosome arrays. Mol Microbiol 32:643–655. doi:10.1046/j.1365-2958.1999.01383.x 10320585

[39] Aubry A, Mougari F, Reibel F, Cambau E. 2017. Mycobacterium marinum. Microbiol Spectr 5. doi:10.1128/microbiolspec.tnmi7-0038-2016 PMC11687479 28387180

[40] Franco-Paredes C, Chastain DB, Allen L, Henao-Martínez AF. 2018. Overview of cutaneous mycobacterial infections. Curr Trop Med Rep 5:228–232. doi:10.1007/s40475-018-0161-7 34164254 PMC8218986

[41] Arttawejkul P, Kongpolprom N. 2018. A case of pulmonary infection caused by Mycobacterium asiaticum: difficulties on diagnostic and therapeutic approaches. Respir Med Case Rep 24:150–152. doi:10.1016/j.rmcr.2018.05.014 29984149 PMC6010662

[42] Yang X, Chen X, Huang J, Chen Y, Zheng W, Chen W, Chen H, Lei S, Li S. 2023. Ultrafast, one-step, label-based biosensor diagnosis platform for the detection of Mycobacterium tuberculosis in clinical applications. ACS Infect Dis 9:762–772. doi:10.1021/acsinfecdis.2c00475 36926845

[43] Wu T, Shen C, Zhao Z, Lyu M, Bai H, Hu X, Zhao J, Zhang R, Qian K, Xu G, Ying B. 2024. Integrating paper-based microfluidics and lateral flow strip into nucleic acid amplification device toward rapid, low-cost, and visual diagnosis of multiple mycobacteria. Small Methods 8:e2400095. doi:10.1002/smtd.202400095 38466131

[44] Thapa J, Maharjan B, Malla M, Fukushima Y, Poudel A, Pandey BD, Hyashida K, Gordon SV, Nakajima C, Suzuki Y. 2019. Direct detection of Mycobacterium tuberculosis in clinical samples by a dry methyl green loop-mediated isothermal amplification (LAMP) method. Tuberculosis (Edinb) 117:1–6. doi:10.1016/j.tube.2019.05.004 31378262

[45] Kapalamula TF, Thapa J, Hayashida K, Chizimu J, Tanomsridachchai W, Nyenje ME, Mkakosya R, Nakajima C, Suzuki Y. 2023. Direct detection of Mycobacterium bovis by a dry loop-mediated isothermal amplification assay in cattle samples collected during routine abattoir examination in Malawi. J Vet Diagn Invest 35:307–310. doi:10.1177/10406387231164596 37029660 PMC10185984

[46] Hayashida K, Kajino K, Hachaambwa L, Namangala B, Sugimoto C. 2015. Direct blood dry LAMP: a rapid, stable, and easy diagnostic tool for Human African Trypanosomiasis. PLoS Negl Trop Dis 9:e0003578. doi:10.1371/journal.pntd.0003578 25769046 PMC4358998

