# Supplementary material for: Development of a multiplex loop-mediated isothermal amplification (LAMP) method for differential detection of Mycobacterium bovis and Mycobacterium tuberculosis by dipstick DNA chromatography
Source: Microbiol Spectr. 2025 Apr 30;13(6):e02421-24. doi: 10.1128/spectrum.02421-24 (PMC12131812; doi:10.1128/spectrum.02421-24)

Supplementary Table 2. Detection rates of multiplex LAMP-PAS

| DNA /tube | MTBC (H37Rv) | | *M. bovis* (BCG Tokyo) | | | |
|---|---|---|---|---|---|---|
| | + | - | both + | 16S + | RD4 + | both - |
| 500pg | 7/7 | 0/7 | 4/4 | 0/4 | 0/4 | 0/4 |
| 50pg | 10/10 | 0/10 | 7/7 | 0/7 | 0/7 | 0/7 |
| 5pg | 15/15 | 0/15 | 17/17 | 0/17 | 0/17 | 0/17 |
| 2pg | 7/7 | 0/7 | 8/8 | 0/8 | 0/8 | 0/8 |
| 1pg | 7/7 | 0/7 | 8/8 | 0/8 | 0/8 | 0/8 |
| 500fg | 21/21 | 0/21 | 15/18 | 1/18 | 2/18 | 0/18 |
| 200fg | 12/14 | 2/14 | 4/8 | 4/8 | 0/8 | 0/8 |
| 100fg | 9/14 | 5/14 | 4/8 | 3/8 | 0/8 | 1/8 |
| 50fg | 10/19 | 9/19 | 1/12 | 4/12 | 2/12 | 5/12 |

positive (+) or negative (-) number / repeat number

both + or both - : both of the 16S band and RD4 band were positive (+) or negative (-)

16S + : 16S band was positive and RD4 band was negative

RD4 + : RD4 band was positive and 16S band was negative

**Supplementary Figure.** Mapping of MTBC-16S rRNA LAMP primers on a multiple alignment of NTM 16S rRNA gene sequences

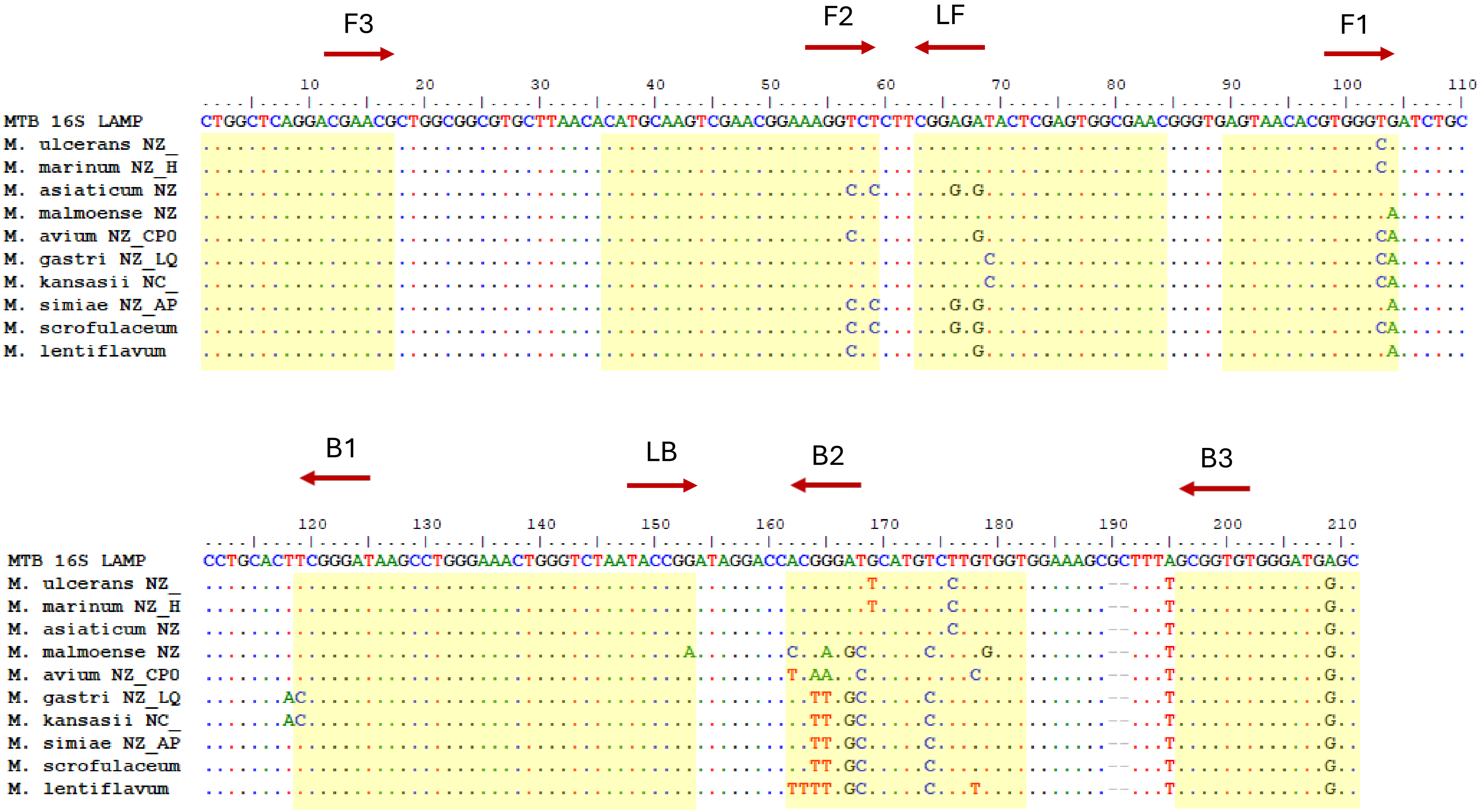

Supplement: Supplemental material — Sensitivity results and sequence alignment. [file spectrum.02421-24-s0001.pdf]
